# Neurosurgical management of pineal lesions: Insights from a single-center study of 27 cases

**DOI:** 10.37796/2211-8039.1700

**Published:** 2026-03-01

**Authors:** David Montesinos Contreras, Walter Stummer, Maryam Khaleghi Ghadiri

**Affiliations:** Klinik für Neurochirurgie, Münster University, 48149, Münster, Germany

**Keywords:** Pineal gland tumors, Pineal region, Lesion, Brain, Cancer, Tumor surgery

## Abstract

**Introduction:**

Pineal lesions are rare and surgically challenging due to their deep location, histological diversity, and potential malignancy. The complexity of the pineal region anatomy and the diverse pathological spectrum contribute to the lack of standardized treatment strategies, making appropriate management controversial.

**Aim:**

This study aimed to evaluate the surgical outcomes of patients with pineal tumors or cysts and to assess the impact of different surgical approaches, particularly the extent of resection, on progression-free survival.

**Methods:**

We conducted a retrospective analysis of 27 patients treated for pineal tumors or cysts at our institution between 2010 and 2020. Data regarding clinical presentation, surgical technique, extent of resection, pathological diagnosis, and follow-up outcomes were collected and analyzed. Surgical outcomes were compared between patients undergoing biopsy and those receiving varying degrees of tumor resection.

**Results:**

Our analyses suggest that total resection could be the most effective approach for reducing the risk of tumor recurrence. Furthermore, our findings indicate that histological subtype is a statistically significant predictor of progression-free survival in patients.

**Conclusion:**

Our findings suggest that achieving a greater extent of resection, when safely feasible, may contribute to improved long-term outcomes. These results emphasize the need for larger, multicenter studies to further optimize treatment strategies for these complex lesions.

## 1. Introduction

The pineal gland, a small pinecone-shaped endocrine structure, is located in the epithalamus region within the brain. The pineal gland borders crucial brain areas, including the thalamus, superior colliculus, and quadrigeminal plate [1]. The two main pathologies of the pineal gland are tumors and cysts. The cell origin of pineal tumors is diverse with over 17 different cell types [2,3]. Pineal tumors are rare and comprise less than 1 % of central nervous system tumors in adults and 3–8 % in children [4–7]. There is a potential ethnic variation in susceptibility to pineal tumors. These tumors have been reported to exhibit a higher prevalence within the Asian population [5].

The most common pineal tumors are germ cell tumors (GCTs), such as germinoma, non-germinomatous germ cell tumors, and teratoma, which account for 50–75 % of cases. This is followed by pineal parenchymal tumors, like pineocytoma and pineoblastoma, comprising 15–30 % of cases [2]. These tumors can metastasize to other areas, with GCTs, ependymoma, and pineal parenchymal tumors showing a higher tendency for metastases. Each tumor type exhibits unique biological behaviors that influence invasiveness and aggressiveness, thereby forming treatment strategies [2,8,9]. GCTs are common in children and adolescents and are often responsive to radiation and chemotherapy, especially for germinomas radiotherapy and chemotherapy are the most efficient therapy. [10–16]. Pineocytoma, a typically low-grade tumor, is more prevalent in individuals aged 30 to 60 [10,17,18]. On the other hand, pineoblastomas are aggressive high-grade tumors that often need a combination of treatment with surgery, radiation, and chemotherapy [19,20]. Pineal gland cysts are typically benign and often discovered incidentally during imaging examinations [21].

Symptoms of pineal gland tumors vary widely, ranging from headaches and nausea to focal neurological disturbances, such as Parinaud syndrome. Obstructive hydrocephalus due to pineal tumors may manifest with headaches, fatigue, nausea, or memory problems, while compression of the brain stem can result in diplopia and eye movement disorders. Hormonal imbalances could occur following compression of the pituitary gland or in conjunction with GCTs. Spinal tumor dissemination may cause back pain and loss of spinal nerve function [22,23]. In most patients, pineal cysts are asymptomatic. In some cases, however, larger cysts can exert pressure on surrounding brain structures, leading to headaches, visual disturbances, or other neurological symptoms [2,24–26].

The treatment of pineal tumors or cysts depends on various factors, including the size, location, the tumor entity, the age of the patient, comorbidities, and symptoms presented by the patient [8,27,28]. Asymptomatic patients, especially those with pineal cysts, typically do not need immediate treatment and can be monitored over time. However, symptomatic patients may benefit from a range of interventions, including medication, surgery, radiation therapy, chemotherapy, or a combination of these approaches [29,30]. A biopsy or resection can be performed to histologically confirm the tumor’s characteristics and determine its malignancy status [21]. Importantly, the treatment of pineal tumors requires a multidisciplinary approach involving neurosurgeons, pediatricians, neurologists, oncologists, and radiotherapists. Current investigations aim to optimize diagnostic methods, refine therapy management, and understand the molecular basis of the tumors [25]. The present study aims to analyze various surgical approaches and their impact on the outcomes of patients with pineal tumors or cysts. The objective is to identify the most effective treatment options for this rare brain disease and implement them for the benefit of future patients.

## 2. Methods

A longitudinal, retrospective analysis of all patients with pineal gland lesions at our institution between 2010 and 2020 was performed. The data were obtained from the internal hospital information system. This study was approved by the ethics committee of the WestphaliaLippe Medical Association (ethics approval number 2023-549-f-S). Patients who did not receive follow-up therapy at Münster University Hospital and those with incomplete data sets were excluded from the study. All data were anonymized by data protection guidelines. Dependent and independent variables and potential confounders were identified and discussed intensively among the authors.

The statistical analysis was performed with IBM SPSS Statistics, version 29.0 (IBM Corp, Armonk, NY, USA). Parameters and settings were set according to the recommendations of the software. The significance level for hypothesis testing was set at α = 0.05. A p-value <0.05 was considered statistically significant. A normal distribution of the data and independence of the variables were assumed. Fisher’s Exact Test was used to calculate correlations between categorical variables among small sample sizes, and the Log Rank Test based on time-to-event data was used for the calculation of overall survival and progressionfree survival. Due to the small sample size, generalizability may be limited.

## 3. Results

**Fig. 1:**
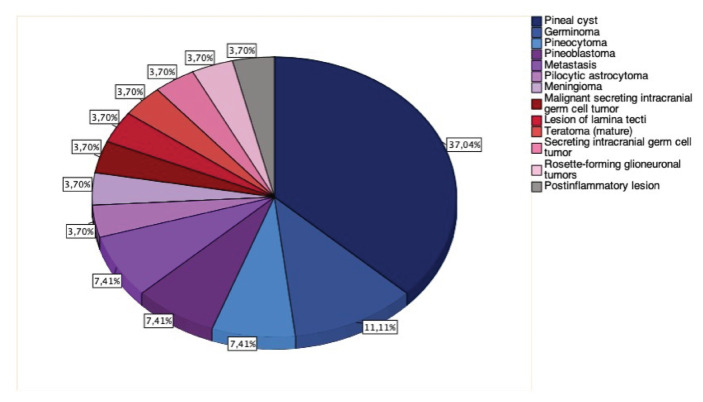
Diagnosis of various forms of pineal lesions among 27 patients.

We retrospectively identified 27 patients diagnosed with pineal gland pathologies treated at the Neurosurgery Department of Münster University Hospital between 2010 and 2020. These included pineal tumors (n = 15), pineal cysts (n = 10), an inflammatory lesion (n = 1), and a lesion of the lamina tecti (n = 1). Among the patients with pineal gland tumors, the spectrum of tumor types was diverse: germinoma (n = 3), metastases (n = 2), pine-oblastoma (n = 2), pineocytoma (n = 2), malignant secreting intracranial GCT (n = 1), meningioma (n = 1), pilocytic astrocytoma (n = 1), rosette-forming glioneuronal tumor (n=1), secreting intracranial GCT (n = 1), and mature teratoma (n = 1). A pathological finding from an endoscopic biopsy in one case indicated a post-inflammatory lesion. Radiomorphological studies suggested a lamina tecti lesion in another patient (Fig. 1).

The mean age of all patients was 28.6 ± 21 years (median 20, range 3–69). In the group of patients with pineal cysts, the mean age was 27.9 ± 12.9 years (median 26, range 13–56). For patients with pineal tumors, the mean age was 29 ± 24.9 years (median 16, range 3–69). Out of the total 27 patients, 14 were male (51.9 %) and 13 were female (48.1 %). Within the pineal cyst group, 1 patient was male (10.0 %) and 9 were female (90.0 %). In the group with pineal tumors, 13 patients were male (86.7 %) and two were female (13.3 %).

Patients presented with a variety of symptoms that prompted their visits to the clinic. Headaches were reported by 17 patients (63%), while an ophthalmologic consultation was registered for 19 patients (70.4 %). Furthermore, six patients (22.2 %) presented with initial symptoms consistent with Parinaud syndrome (dorsal midbrain syndrome) and seven patients (25.9 %) reported experiencing other visual disturbances, including convergence weakness, trochlear nerve palsy, and papilledema. Hydrocephalus, due to cerebrospinal fluid (CSF) accumulation, was observed in 19 patients (70.4 %), 10 of them (37 %) received a shunt. Spontaneous improvement of hydrocephalus occurred in 7 patients following tumor resection without the need for shunt placement. In these cases, the resection contributed to pressure relief and/or the restoration of CSF flow. Tumor markers were identified in the CSF of six out of the 17 patients with pineal tumors. β-human chorionic gonadotropin (β-hCG) alone was detected in three patients, two had a germinoma, one had a pineoblastoma, and 1 from a pineoblastoma. Furthermore, β-hCG was found together with α-fetoprotein (AFP) in two patients, one with a teratoma and one with a malignant secreting intracranial GCT. AFP was detected as a tumor marker in the CSF of one patient with a secreting intracranial GCT.

**Fig. 2:**
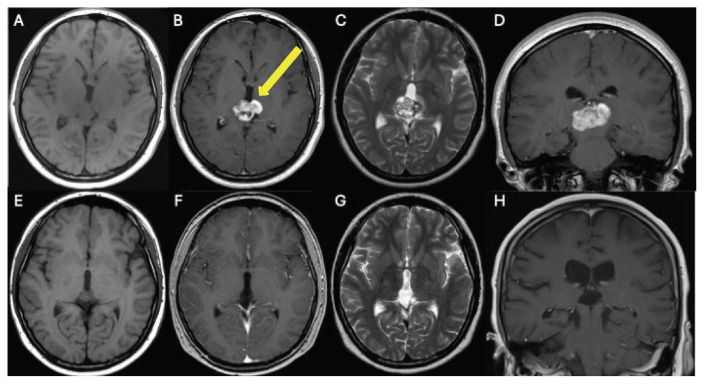
MRI of one patient with a pineal gland tumor. **(A)** Axial T1 sequence without contrast medium before treatment. An exophytic growth pattern of the tumor can be seen, there is no hydrocephalus recognizable. **(B)** Axial T1 sequence with contrast medium before radiation. **(C)** Axial T2 sequence with contrast medium before radiation. **(D)** Coronal T1 sequence with contrast medium before radiation. **(E)** Axial T1 sequence without contrast medium after radiation. **(F)** Axial T1 sequence with contrast medium after resection. **(G)** Axial T2 sequence with contrast medium after resection. **(H)** Coronal T1 sequence with contrast medium after resection.

**Fig. 3:**
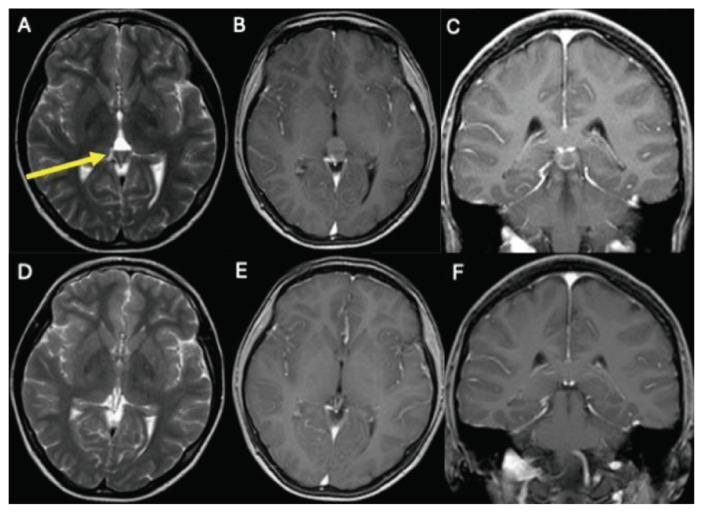
MRI of one patient with a haemorrhaged pineal cyst. A mirror formation is recognisable. **(A)** Axial T2 sequence with contrast medium before resection. **(B)** Axial T1 sequence with contrast medium before resection. **(C)** Coronal T1 sequence with contrast medium before resection. **(D)** Axial T2 sequence with contrast medium after resection **(E)** Axial T1 sequence with contrast medium after resection **(F)** Coronal T1 sequence with contrast medium after resection.

Surgical approaches include sampling, resection, third ventriculostomy, shunt implantation, and Rickham reservoir insertion. Eighteen out of 27 patients with pineal lesions underwent surgical treatment. At our hospital, surgery was performed on seven out of 10 patients with pineal cysts, all of whom underwent total resections. Four patients were operated on through an infratentorial-supracerebellar approach, while three patients were operated on using a suboccipital approach. All patients were symptomatic with neurological disturbances; four had a cyst larger than 2 cm in diameter. Three out of 10 patients with pineal cysts are under regular monitoring as part of a watchful waiting approach. Figs. 2 and 3 show imaging of two patients with pineal tumor and pineal cyst before and after surgery, respectively.

Of the 17 patients with various pineal pathologies, 11 received surgical treatment, with six undergoing additional procedures before resection, including third ventriculostomy, sampling, external ventricular drain placement, shunt placement, and Rickham reservoir implantation. Among these, five patients had total resections, while six had subtotal resections. Nine patients underwent resection without a prior biopsy, while two had biopsies followed by resection. Patients with pineal lesions were operated on using either a suboccipital approach or an infratentorial-supracerebellar approach. Among the 18 patients who underwent surgery, 10 had an infratentorial approach, including four with cysts and six with other pineal lesions. Furthermore, eight patients underwent a suboccipital approach, including three with cysts and five with other lesions.

Regarding adjunctive treatments for pineal lesions, seven patients received postoperative radiochemotherapy. Two patients with GCTs were given preoperative radiochemotherapy. One patient with a mature teratoma underwent preoperative chemotherapy followed by postoperative radiochemotherapy. Moreover, two patients received postoperative irradiation; one for a glioneural tumor and the other for a meningioma.

Among the patients with tumors in the pineal region, 12 underwent radiotherapy, and 10 received additional chemotherapy. The main chemotherapy regimens employed for these patients with pineal tumors included the SIOP-CNS-GCT-96 protocol and HIT-SKK 2000. None of the patients with pineal cysts experienced a recurrence, whereas recurrence of tumors was observed in seven patients with pineal tumors. Among these seven patients, four cases underwent subtotal resection, and three patients underwent total resection. Complications were reported in seven cases including hygroma, CSF cushion, CSF leak due to bone cement loosening (one case each), two instances of shunt revision, postoperative visual impairment, and permanent headache.

Follow-up assessments of these patients revealed that four patients experienced spinal dissemination of the primary tumor. Moreover, one patient displayed abdominal metastases from a cerebral hindgut tumor, while two others exhibited brain metastases from different primary tumors. During the follow-up of patients with pineal tumors, three patients were confirmed deceased.

Our analysis of the correlation between the extent of resection (no resection, subtotal resection, and total resection) and tumor recurrence indicated a trend suggesting that total resection could be the most effective approach for reducing the risk of recurrence, although further studies are needed to confirm this potential association (*P* = 0.056). Furthermore, the application of radiochemotherapy showed an inverse correlation with tumor recurrence (*P* = 0.024). Moreover, a negative significant correlation between hydrocephalus and shunt placement was observed (*P* = 0.012). Furthermore, our analysis revealed that the histological subtype has a statistically significant effect on progression-free survival (*P* < 0.001). Other factors, including operative method (*P* = 0.282), WHO grade (*P* = 0.557), and tumor diameter (*P* = 0.813), did not show significant correlation with both progression-free survival (median: 3402 ± 1572 days). None of the above-mentioned factors showed a statistically significant impact on overall survival. Although the study is constrained by a small sample size, a significant rate of censoring, and a limited number of events that together diminish statistical power, emerging trends indicate that additional research in larger, more sufficiently powered studies is necessary.

## 4. Discussion

Pineal gland tumors and cysts present a significant surgical challenge due to their deep brain location. The primary treatment for pineal tumors, when feasible, is surgery. The objectives of surgery are: *(i)* to obtain tissue samples for accurate tumor diagnosis and *(ii)* to remove as much of the tumor as possible while reducing the risk of complications. Several surgical approaches are available, including the infratentorial-supracerebellar approach, the occipital transtentorial approach, and the suboccipital approach. Minimally invasive procedures, such as endoscopic third ventriculostomy, are also used for the removal of pineal lesions. Each method has distinct advantages and disadvantages, which are detailed below [1,7,31–35]. The infratentorial-supracerebellar approach provides access to the pineal region from below, allowing direct access to lesions in the posterior part of the pineal gland. This approach minimizes brain retraction and does not significantly impede the venous return from the upper cerebellar surface to the tentorium. However, this approach is not suitable if significant veins connect the cerebellum to the tentorium or if the angle of the straight venous sinus is steep [1,31,36]. In contrast, the suboccipital approach offers a direct, posterior route to the pineal gland by removing a portion of the occipital bone. This surgical approach is especially effective for accessing lesions near the midbrain tectum [1,34,35]. For our patients, the surgical approaches considered were infratentorial-supracerebellar and suboccipital. These approaches showed no significant differences in terms of complications and tumor recurrence. If resection is not possible, a stereotactic or endoscopic biopsy provides an alternative to obtain samples for histopathological investigations. This enables an accurate diagnosis and facilitates the assessment of further treatment strategies. In cases of complex tumors, a combination of the afore-mentioned surgical methods and approaches may be required to minimize morbidity. A watchful waiting strategy is mostly used for patients who are not seriously ill [8,9,29,34–37]. However, it should be noted that the non-randomized allocation of treatment (biopsy vs. resection) introduces potential selection bias, as surgical decisions may have been influenced by factors such as tumor size, location, or patient condition. This limitation could affect the comparability of outcomes across different groups and should be taken into account when interpreting our results.

Our findings indicate a possible link between the extent of tumor resection and the recurrence of pineal tumors, pointing to the need for further evaluation to confirm a definitive relationship. Subtotal resection is more likely to lead to recurrence compared to complete resection, consistent with findings in other tumor studies. Furthermore, our study indicates a correlation between the occurrence of hydrocephalus and the need for shunt placement, aligning with guideline-compliant procedures for managing occlusive hydrocephalus [38]. In keeping with our results, a retrospective study of 25 patients with tumors in the pineal region, mainly GCTs and gliomas, three-dimensional exoscopic resection resulted in a 76 % gross total resection rate, with effective hydrocephalus relief and minimal complications. At followup, 22 of the 25 patients showed no tumor recurrence [39]. In another retrospective evaluation of 61 patients with pineal tumors treated using different surgical approaches, including supracerebellar infratentorial, posterior transfalcine interhemispheric, and occipital transtentorial, complete tumor resection was achieved in 55 % of patients. Seventeen patients had hydrocephalus requiring ventricular drainage, and postoperative adjuvant therapy was administered in cases of glial tumors and pineoblastomas. Transient Parinaud’s syndrome occurred in 30 % of patients but resolved within two months. This study suggests the importance of surgical planning, anesthetic management, and proper patient positioning to achieve favorable outcomes [40]. Moreover, in a prospective study of 32 patients with pineal lesions (20 tumors and 12 cysts), surgical treatment through the infratentorial supracerebellar approach resulted in significant improvements in neurological symptoms in 75 % of tumor patients and all cyst patients, with only minor morbidity. Total resection significantly reduced the need for permanent shunting compared to subtotal resection, with five patients experiencing recurrence [41]. In a 30-year study involving 151 children diagnosed with pineal tumors, resection was mainly performed using the supracerebellar infratentorial approach. Total resection was achieved in 64 % of cases and significantly associated with improved 5-year overall survival, especially in patients with GCTs. In line with our findings, the efficacy of resection differed based on histological type, with total resection being significantly associated with higher overall survival rates [42]. In contrast, a 17-year retrospective study of 43 pediatric patients also identified germinomas and pineoblastomas as the most common diagnoses, but found that while endoscopic third ventriculostomy became the preferred method for managing hydrocephalus, often combined with biopsy, open resection was linked to increased rates of complications without a corresponding survival benefit [43]. These discrepancies between these two studies point to the importance of factors such as surgical strategy and histological distribution in patient outcomes and prognosis.

Several biomarkers were identified in some of our patients with pineal tumors. Some types of pineal tumors, particularly non-germinomatous GCTs, and immature teratomas, produce specific proteins such as AFP and β-hCG, which can be used as diagnostic markers. These markers, measurable in serum and CSF, help diagnose, monitor treatment response, and track disease recurrence. Yolk sac tumors, embryonal carcinomas, and immature teratomas primarily produce AFP, while choriocarcinomas and embryonal carcinomas produce β-hCG. Germinomas and mature teratomas do not produce these markers. Carcinoembryonic antigen (CEA) is not typically associated with pineal tumors [19,42–45].

Magnetic resonance imaging (MRI) provides images that allow for the precise visualization of the pineal region and surrounding brain structures, distinguish between different types of pineal lesions, and help in assessing the size, extent, and impact of the lesion on adjacent areas. Furthermore, MRI provides essential anatomical details that are important for planning surgical approaches, determining the possibility of resection, minimizing potential complications, and ongoing monitoring of pineal lesion changes over time. An MRI with and without contrast of the entire brain and spine is essential. Pineal cysts are commonly benign and typically exhibit contrast enhancement in the cyst wall. However, irregularities in the cyst wall, such as nodules, may suggest malignancy [10,13,16,46–49].

While our descriptive analyses provide insights into observed trends and correlations among different clinical parameters, the generalizability and inferential power of this study are limited by small sample size. The retrospective and single-center design further limits the applicability of the findings to broader populations. These limitations underscore the need for larger, multi-center prospective studies to confirm our findings and to enable more robust conclusions for the wider patient population.

## 5. Conclusion

In our study, we observe consistent patterns indicating that resection may offer superior outcomes compared to sampling alone, as evidenced by improved progression-free and overall survival rates. For highly proliferative and rapidly growing tumors, supplementary radiotherapy and potentially chemotherapy may prove beneficial in reducing recurrence risk. To enhance diagnostic accuracy, optimize therapeutic strategies, and deepen our understanding of the molecular mechanisms underlying these tumors, further research with larger patient cohorts is needed to validate our findings. The treatment strategy is selected depending on the tumor characteristics, the age of the patient, and the patient’s comorbidities. Surgical interventions should achieve a safe and complete resection while preserving neurological function.
